# Predicting Changes in Macrophyte Community Structure from Functional Traits in a Freshwater Lake: A Test of Maximum Entropy Model

**DOI:** 10.1371/journal.pone.0131630

**Published:** 2015-07-13

**Authors:** Hui Fu, Jiayou Zhong, Guixiang Yuan, Chunjing Guo, Qian Lou, Wei Zhang, Jun Xu, Leyi Ni, Ping Xie, Te Cao

**Affiliations:** 1 Jiangxi Provincial Key Laboratory of Water Resources and Environment of Poyang Lake, Jiangxi Institute of Water Sciences, Nanchang, China; 2 Donghu Experimental Station of Lake Ecosystems, State Key Laboratory of Freshwater Ecology and Biotechnology, Institute of Hydrobiology, The Chinese Academy of Sciences, Wuhan, China; Università di Genova, ITALY

## Abstract

Trait-based approaches have been widely applied to investigate how community dynamics respond to environmental gradients. In this study, we applied a series of maximum entropy (maxent) models incorporating functional traits to unravel the processes governing macrophyte community structure along water depth gradient in a freshwater lake. We sampled 42 plots and 1513 individual plants, and measured 16 functional traits and abundance of 17 macrophyte species. Study results showed that maxent model can be highly robust (99.8%) in predicting the species relative abundance of macrophytes with observed community-weighted mean (CWM) traits as the constraints, while relative low (about 30%) with CWM traits fitted from water depth gradient as the constraints. The measured traits showed notably distinct importance in predicting species abundances, with lowest for perennial growth form and highest for leaf dry mass content. For tuber and leaf nitrogen content, there were significant shifts in their effects on species relative abundance from positive in shallow water to negative in deep water. This result suggests that macrophyte species with tuber organ and greater leaf nitrogen content would become more abundant in shallow water, but would become less abundant in deep water. Our study highlights how functional traits distributed across gradients provide a robust path towards predictive community ecology.

## Introduction

Trait-based approaches have been widely applied to investigate how community dynamics respond to environmental gradients [1–4]. The trait-based community assembly processes (i.e., habitat filtering and niche differentiation) may predict successively the species’ presence or absence in a local community [5,6], whereas will not well explain its dominance or rarity (i.e., relative abundance). However, a trait-based approach assumes that individuals with traits that confer high performance in a given environment will be able to sequester a greater proportion of limited resources for growth and reproduction, resulting in greater relative abundance within community [4,5]. According to this assumption, Cornwell and Ackerly [7] reported that there were significant links between plant traits and abundance, implying a potential trait-based processes affecting species relative abundance. Shipley et al. [8] proposed a maximum entropy (maxent) model that can potentially link traits to community composition at the species level by predicting relative abundances of each species in a community using community-weighted mean (CWM) traits as constraints. The maxent model is suggested as a mathematical translation of the notion of trait-based environmental filtering; it is a mathematical consequence of non-random processes (i.e., natural selection) acting on all individuals in a local community [9]. The maxent model provided a quantitative framework: if the individuals of species with favorable traits in a specific habitat become more abundant, species sorting processes will constrain CWM traits values in a local community and such CWM traits will then possess information that is translated into the maximum entropy probabilities [8–11]. CWM traits can vary predictably along environmental gradients, allowing us to predict how the relative abundances of species possessing these traits will vary along this gradient [12]. Despite the remarkably strong predictive ability (> 90%) of maxent model on the variance in relative abundance given the observed CWMs [8,12,13], the robust nature of maxent model in predicting changes in community structure along environmental gradients remained to be tested in various types of plant communities based on predicted CWM traits.

In freshwater habitats, macrophyte communities are strongly influenced by water depth [6,11,14–17]. Recently, Fu et al. [18] found that macrophyte community biomass showed a hump-shaped pattern along water depth gradient, while biomass of most macrophyte species in the community displayed linear pattern along the gradient. Remarkably, CWM traits were the most important predictors affecting macrophyte community biomass, although the strength and direction of those effects depended on selected trait [18]. CWM traits differed largely in their responses to water depth gradient, which may mirror the relative changes of abundance of particular species possessing dominant traits along the gradient. Using a trait-based approach, Fu et al. [6] demonstrated that the significant non-random processes (i.e., habitat filtering and niche differentiation) shape the functional trait distribution (e.g., CWM traits) within communities along environmental gradients. Both processes of habitat filtering and niche differentiation affected the functional responses of macrophyte communities associated with different sets of traits in significant different patterns along water depth gradient. The deterministic assembly processes acting on functional composition of macrophyte community in a local scale would largely inspire us to incorporate maxent models to predict species relative abundance. Our recent works have indicated the variations of CWM traits reflect the significant trends in the effects of the deterministic assembly processes on species’ presence and thus community abundance along the water depth gradient [6]. However, what are the degrees of different functional traits in determining macrophyte species abundance and how does the importance of different traits vary along the gradient have yet to be studied [19,20].

In this study, we applied a series of maxent models to unravel the processes governing macrophyte community structure along water depth gradient in a freshwater lake. We examine the robustness of maxent model in predicting species relative abundance with observed or fitted (as predicted by water depth gradient) CWM trait values as constraints. Based on the intrinsic concept of maxent model, that natural selection occurring between individuals of different species, we hypothesized that: (1) the changes in macrophyte community structures can be predicted by maxent model with CWM traits fitted from water depth gradient as constraints, because there were significant effects of non-random processes on CWM traits and thus community abundance [6,18,21]; (2) functional traits differ in their importance in determining species relative abundance, because macrophyte species exhibit significant different performance along different functional niches [6]; and (3) the relative importance of different functional traits in determining species relative abundance will vary along water depth gradient, because the strength and direction of non-random effects on different traits vary significant along the gradient [6].

## Materials and Methods

Our study complies with the current laws of China and with international rules. No specific permissions were required for the described field studies; all samples were obtained from publicly accessible waters and the studied species are not endengered or protected.

### Field sampling designs and data collecting

**Fig 1:**
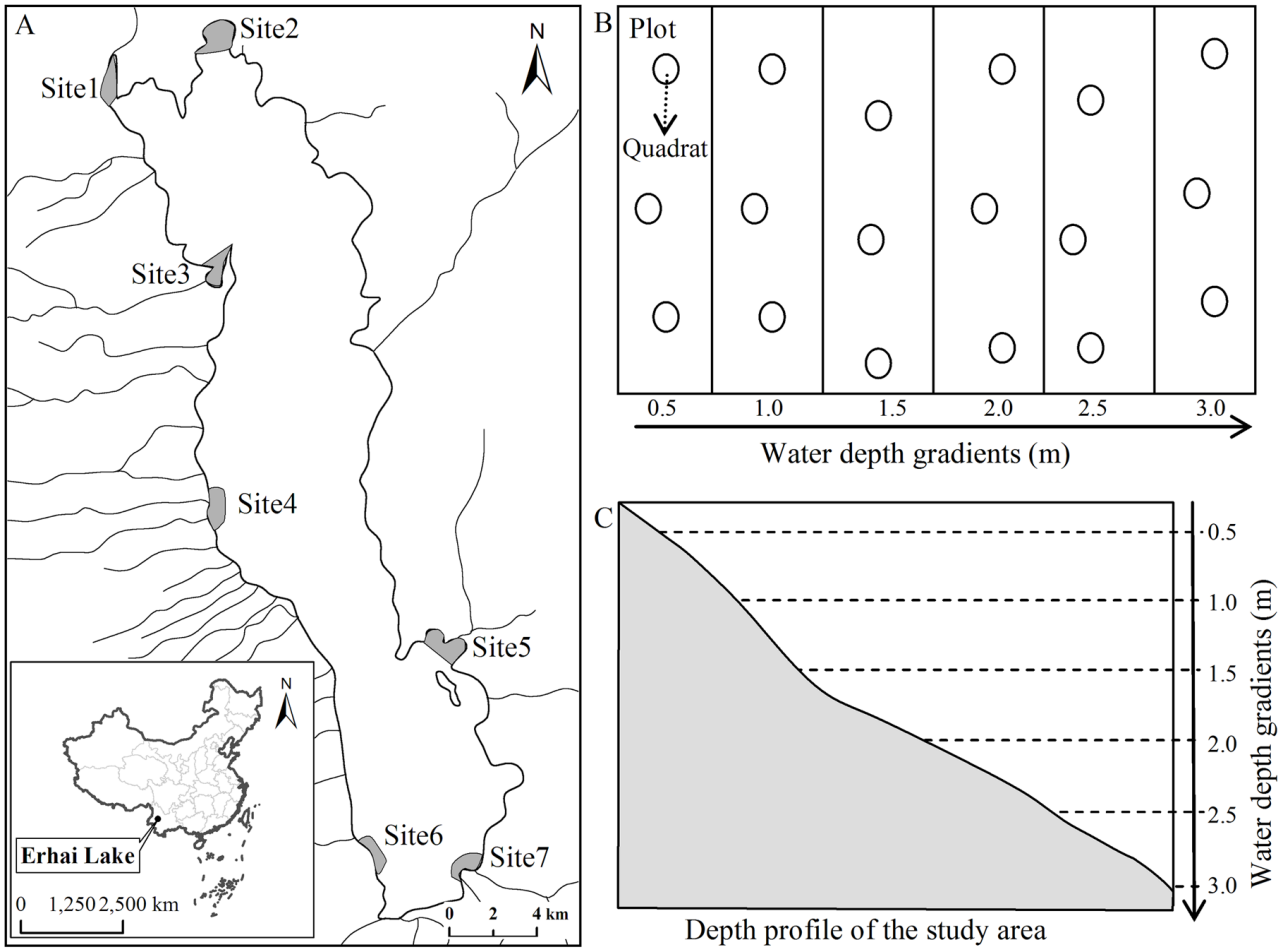
The study location and scheme of the sampling design. (a) The study was carried out in Erhai Lake (25°52'N, 100°06'E) in Yunnan Province, China. We sampled macrophyte communities in forty-two 25 m^2^ plots at seven sites. At each site, six 5 × 5 m plots were located along the water depth gradient at 0.5 m intervals from 0 m to 3.0 m depth (b, c). Within each 25 m^2^ plot, we used three 0.2 m^2^ quadrats for this analysis (b).

The study was carried out in Erhai Lake (25°52'N, 100°06'E) in Yunnan Province, China and the details of sampling designs were described in our previous studies [6,18]. The study location and sampling scheme were showed in Fig 1. The original abundance and trait datasets were described in details in Fu et al. [18] and Fu et al. [6]. The abundance of macrophyte community was estimated in forty-two 25 m^2^ plots at seven sites. At each site, six 5 × 5 m plots were located along the water depth gradient at 0.5 m intervals from 0 m to 3.0 m depth. Within each 25 m^2^ plot, we used three 0.2 m^2^ quadrats for this analysis and we sampled a total of 126 quadrats. The mean abundances of each species across water depth gradient were showed in Table 1, and the mean trait values of each species were presented in our previous studies (see S1 Table listed in the Supporting Information of ref. [18]). We measured (or collected date from literature) 16 key functional traits (Table 2) on all 17 macrophyte species following standardized protocols [21]. The 16 functional traits are: mean Julian flowering dates, flowering duration, floating leaf, perennial growth form, tuber, specific leaf area, leaf dry mass content, lamina thickness, leaf carbon [C] content, leaf nitrogen [N] content, leaf carbon/nitrogen [C/N] ratio, ramet size, shoot height, stem dry matter content, stem diameter and rooting depth. We sampled a total of 1513 individuals for the measurements on functional traits.

**Table 1:** The mean abundance (kg dry weight m^-2^) of 17 macrophyte species across the water depth gradient.

Species	Water depth (m)					
	0.5	1.0	1.5	2.0	2.5	3.0
*Potamogeton praelongus Wulf*	0.011	0.000	0.000	0.000	0.000	0.000
*Potamogeton pectinatus*	0.022	0.002	0.014	0.019	0.000	0.001
*Potamogeton perfoliatus*	0.052	0.058	0.023	0.036	0.052	0.000
*Najas marina*	0.003	0.000	0.001	0.000	0.000	0.000
*Potamogeton lucens*	0.051	0.220	0.072	0.171	0.090	0.074
*Hydrilla verticillata*	0.197	0.071	0.089	0.070	0.065	0.038
*Myriophyllum spicatum*	0.093	0.094	0.038	0.025	0.011	0.024
*Potamogeton maackianus*	0.039	0.039	0.176	0.279	0.273	0.301
*Ceratophyllum demersum*	0.020	0.017	0.023	0.050	0.055	0.010
*Vallisneria natans*	0.116	0.075	0.199	0.182	0.259	0.403
*Polygonum amphibium L*.	0.020	0.083	0.005	0.000	0.000	0.000
*Trapa natans L*.	0.096	0.084	0.188	0.061	0.046	0.020
*Potamogeton malaianus*	0.148	0.145	0.134	0.092	0.076	0.101
*Potamogeton intortifolius*	0.026	0.053	0.018	0.008	0.071	0.027
*Hydrocharis dubia (Bl*.*) Backer*	0.000	0.016	0.000	0.000	0.000	0.000
*Potamogeton distinctus A*.*Bennett*	0.066	0.024	0.000	0.000	0.000	0.000
*Nymphoides peltatum(Gmel*.*)O*.*Kuntze*	0.041	0.019	0.020	0.007	0.000	0.000

**Table 2:** List of 16 plant functional traits studied for 17 macrophyte species. The mean lambda values derived from the maxent models in which traits are standardised to unit variance.

Functional traits	Unit	Characteristic	Function	Mean lambda values
Floating leaf	Ordinal:(1 = no, 2 = yes)	Phenology	Canopy architecture, Space niche in canopy, Light interception.	10.94
Perennial growth form	Ordinal:(1 = no, 2 = yes)	Phenology	Growth time strategy	-1.93
Tuber	Ordinal:(1 = no, 2 = yes)	Phenology	Organ turnover, Growth strategy	-2.23
Mean Julian Flowering Date	Continuous (day)	Phenology	Growth time strategy	27.74
Flowering duration	Continuous (Julian day)	Phenology	Growth time strategy	30.50
Ramet size	Continuous (mg)	Morphology	Growth strategy, Space niche in habitats	7.12
Shoot height	Continuous (cm)	Morphology	Light capture, Competition, Canopy architecture,	22.87
Stem diameter	Continuous (mm)	Morphology	Stem architecture, water uptake strategy	6.76
Specific leaf area	Continuous (m^2^ kg^-1^)	Morphology	Assimilate utilization, Light interception, space niche in canopy	23.81
Leaf dry mass content	Continuous (g g^-1^)	Morphology	Assimilate utilization, Palatability, Decomposability	32.57
Lamina thickness	Continuous (mm)	Morphology	Resources acquisition strategy	6.16
Rooting depth	Continuous (cm)	Morphology	Space niche in soil, Nutrient acquisition strategy	-7.53
Stem dry mass content	Continuous (g g^-1^)	Morphology	Assimilate utilization, Water transport	11.81
Leaf nitrogen content	Continuous (mg g^-1^)	Chemical composition	Photosynthetic capacity, Palatability	29.15
Leaf carbon content	Continuous (mg g^-1^)	Chemical composition	Palatability, Decomposability	-13.37
Leaf carbon/nitrogen ratio	Continuous (g g^-1^)	Chemical composition	Photosynthetic capacity, Palatability	18.95

### Statistical analysis

We used CWM trait values as the constraint in the maxent model to predict the species relative abundance. CWM trait values were used to describe the functional composition of each plot [22] and was calculated as $\sum_{i\;=\;1}^{S}PiTi$, where *P*_*i*_ is the proportional biomass of *i*th species in the community, *T*_*i*_ is the trait values of species *i*, and *S* is the number of species.

The general description of the maxent model has been characterized by Shipley et al [8,12]. The maximum entropy (maxent) predicted values of relative abundance of 17 species in each of 42 plots were obtained using the “maxent” and “maxent.test” function of FD package for R. Macroscopic constraints were based on the CWM trait values (observed or fitted from the general linear models based on water depth gradient) of each plot. Uniform prior distributions were used in all instances. Convergence occurred when the largest change in any single predicted relative abundance between two iteration was < 1×10^7^. Significance tests were performed using permutation test of Shipley [23], also found in FD package for R.

We used two different methods to obtain maxent models containing fewer traits as suggested by Shipley *et al*. [12]. We used a backwards stepwise procedure to calculate the mean absolute lambda coefficients over the 42 plots, sequentially remove the trait having the lowest absolute lambda, and then refit the model with all traits except for this one for models based on observed CWMs (model 1). We fitted linear mixed models relating the observed CWM traits to the water depth gradient and used these to obtain the fitted CWM traits for each plot based only on its environmental conditions. Then, we inputted sequentially traits having the highest *R* ^*2*^ explained by water depth gradient for models based on fitted CWMs (model 2), and the order is: stem dry mass content (*R* ^*2*^ = 0.47), leaf carbon content (*R* ^*2*^ = 0.42), leaf nitrogen content (*R* ^*2*^ = 0.26), flowering duration (*R* ^*2*^ = 0.16), floating leaf (*R* ^*2*^ = 0.13), leaf dry mass content (*R* ^*2*^ = 0.12), lamina thickness (*R* ^*2*^ = 0.1), leaf carbon/nitrogen ratio (*R* ^*2*^ < 0.1), perennial growth form (*R* ^*2*^ < 0.05), stem diameter (*R* ^*2*^ < 0.05), mean Julian flowering dates (*R* ^*2*^ < 0.05). tuber (*R* ^*2*^ < 0.05), specific leaf area (*R* ^*2*^ < 0.05), rooting depth (*R* ^*2*^ < 0.01), ramet size (*R* ^*2*^ < 0.01), shoot height (*R* ^*2*^ < 0.01). A general linear mixed model was applied to assess the variations of species relative abundance along the water depth gradient, with sites as random effects.

The λ-values for each trait that are estimated by the maxent model measure the importance of the trait in determining the predicted relative abundance, holding other traits constant [8,12]. For a given trait, species possessing higher trait values would be more abundant than expected when λ-value > 0, and *vice versa* when λ-value < 0; this trait has no effects on species relative abundance when λ-value = 0 [8,20]. Therefore, we can use the λ-values, derived from the maxent models, to inspect the strength and direction of filtering processes on individuals with a specific trait along water depth gradient during community assembly. To assess the relative importance of a specific trait in determining abundance across the water depth gradient, a general linear mixed model was applied to assess the variations of the λ-values (without standardizing) along the water depth gradient, with sites as random effects.

Furthermore, when traits are standardised to unit variance, we can use the mean λ-value for each trait to identify what extent of different traits in determining species relative abundance. In present study, the λ-values for each trait were either positive or negative across the 42 plots, and thus we only showed the mean λ-value for each trait in Table 2. The greater mean |*λ*|-values of a given traits across 42 plots indicates a stronger effects of this trait on species relative abundance. Prior to analysis, Mean Julian flowering dates, flowering duration, ramet size, shoot height, specific leaf area, leaf dry mass content, stem dry mass content, leaf [C], leaf [N] and leaf [C/N] were log-transformed to reduce the influence of species with large trait values and all traits were then standardised to unit variance. When negative trait values were obtained from the log-transformation, we added |x_min_| to all values for that trait, where x_min_ is the minimum trait value. This translation was necessary for the Improved Iterative Scaling algorithm, which cannot accept negative values [7].

## Results

**Fig 2:**
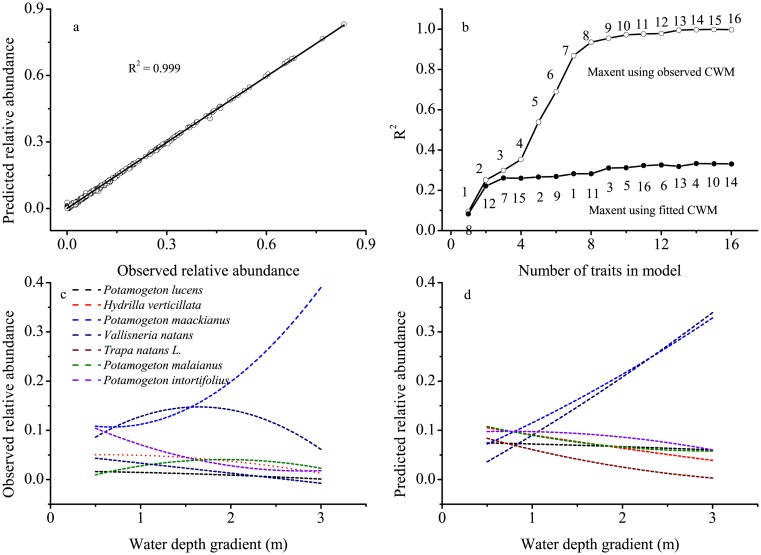
Results of a backward stepwise analysis of maximum entropy (maxent) model using community-weighted mean trait (CWM) constraints. (a) Observed vs. predicted relative abundances of 17 macrophyte species over 42 plots using observed sixteen CWM traits. (b) Relationship between the amount of explained variance of maxent model and the number of traits used in the model. Open circles represent models that used observed CWM traits as constraints, while filled circles represent models that used fitted CWM traits as constraints. Filled circles represent models in which traits were entered into the model in the order at which they are listed from left to right, based on how well they could be predicted from water depth gradient. The numeral codes showed in this chart indicates: 1-lamina thickness, 2-floating leaf, 3-perennial growth form, 4-rooting depth, 5-stem diameter, 6-tuber, 7-leaf nitrogen content, 8-stem dry mass content, 9-leaf dry mass content, 10-ramet size, 11-leaf carbon/nitrogen ratio, 12-leaf carbon content, 13-specific leaf area, 14-shoot height, 15-flowering duration, 16-mean Julian flowering dates. General linear models of observed (c) and predicted (d) relative abundances of seven common macrophyte species presented in Erhai Lake along water depth gradient. The predicted relative abundances used here were generated with the maxent model using the seven community-weighted mean trait constraints that were best predicted from water depth gradient.

Using the observed CWM for all 16 traits, the maxent model estimates predicted 99.8% of the observed variation in the actual relative abundance of these 17 species in this lake (Fig 2a). The maxent predictions of relative abundances in the validation data set were significant with only two traits (i.e., lamina thickness and floating leaf), and predictive ability increased asymptotically with the number of traits used in the model (Fig 2b).

A maxent model using the fitted CWM traits as constraints gave the predicted relative abundances of the 17 species in each plot. When the two traits of best predicted from the depth gradient were included, the models explained 22.1% of the variation in relative abundances that was potentially explicable from water depth gradient (Fig 2b). The predicted relative abundance from these species-specific linear mixed models and maxent models using the best eight CWM traits were similar (Fig 2c and 2d). For example, the relative biomass increased for *Potamogeton malaianus*, *Potamogeton maackianus* and *Vallisneria natans*, but decreased for *Potamogeton intortifolius*, *Potamogeton perfoliatus*, *Hydrilla verticillata* and *Trapa natans*.

**Fig 3:**
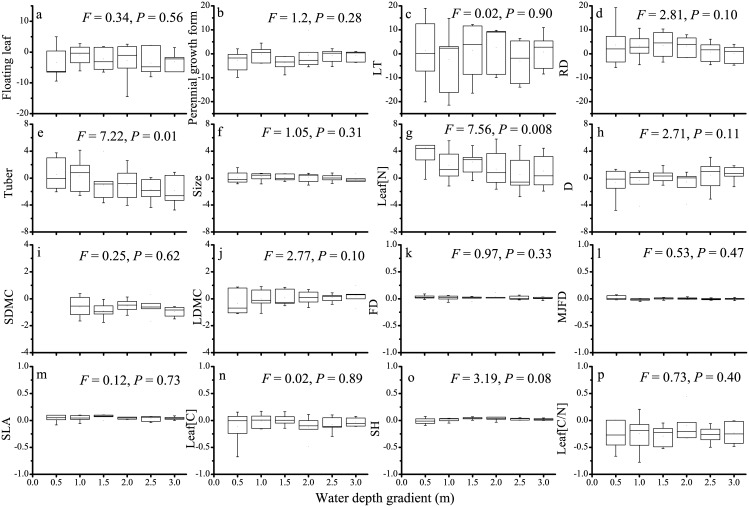
Boxplots showing the differences in λ-values derived the maxent models along water depth gradient for the 16 measured traits of macrophyte species. The *F* and *P* values from linear regression analyses are shown. LT: lamina thickness; RD: rooting depth; Leaf [N]: leaf nitrogen content; D: stem diameter; SDMC: stem dry mass content; LDMC: leaf dry mass content; FD: flowering duration; MJFD: mean Julian flowering date; SLA: specific leaf area; Leaf [C]: leaf carbon content; SH: shoot height; Leaf [C/N]: leaf carbon/nitrogen ratio.

Strongly difference in the relative importance of different functional traits in determining species relative abundance were found, with the mean λ-values (with standardizing) across the 42 plots ranged from -1.93 for mean perennial growth form to 32.6 for leaf dry mass content (Table 2). Significant trends of λ-values (without standardizing) along water depth gradient were detected for tuber (Fig 3e) and leaf nitrogen content (Fig 3g), while marginally significant effects of water depth on λ-values were detected for rooting depth (Fig 3d), stem diameter (Fig 3h), leaf dry mass content (Fig 3j) and shoot height (Fig 3o).

## Discussion

We found highly significant associations between observed relative abundance and plant traits using as few as two traits, > 60% of the variation in relative abundances could be predicted with as few as six traits, and essentially all of the observed variation in relative abundances was captured using all 16 traits. When using CWM traits values fitted from water depth gradients, the maxent model explained no more than 32% of the variations of relative abundances, which supports partially the first hypothesis. This relative lower explanatory power might be attributed to the following reasons. On the one hand, some important functional traits (i.e., branching patterns, root/shoot ratio and internode length) related to water depth were not included into this model [16,17,24]. On the other hand, only one environmental variable might provide not enough information for this predictive model, because macrophyte communities structured in freshwater habitats associating multiple environmental factors [25–28]. Although many other potential important environmental factors (i.e., nutrients and sediment) were not included in this analysis, which may largely promote the predictive ability of fitted CWM, our results demonstrated that water depth played an important structuring role in macrophyte community compositions. The dominant macrophyte species showed a predictable change along water depth gradient, which acts as a filter sorting community average trait values and thus functional strategies within communities. For this point, we can obtain some useful information about the variations in macrophyte community structure from the maxent models based on fitted CWM along environmental gradients, which would have important inspirations for the conservation and management of freshwater ecosystems especially under conditions of water level fluctuation.

In supporting our second hypothesis, the measured traits showed notably distinct importance in predicting species abundances. In present study, leaf dry mass content was the most important trait determining species abundance when the other traits were held constant. Species possessing higher leaf dry mass content usually showed more conserved strategies associated with lower growth rate, greater leaf life-span through extra structural strength, and therefore long-lived leaves can accumulate a greater leaf mass and capture a lot of light in that way. This result suggested that macrophyte species with conservative strategies (e.g., greater positive λ-values for high leaf dry mass content) become more abundant in freshwater environment. In support of this point, our recent studies indicated that macrophyte species favored conservative strategies involved in carbohydrate metabolism in responses to eutrophication in this studied lake [29]. Furthermore, mean Julian flower data and flowering duration were also important in predicting species abundance. This result suggests that species flowering later and then lasting longer time would become more abundant. For most marophyte species, clonal growth is a favorable way of expansion and biomass accumulation in freshwater habitats [30], while the processes of expansion and biomass accumulation would be slown down during flowering. Macrophyte species flowering later can have longer clonal growth time for accumulating abundance, and longer flowering duration can produce more seeds for the regenerations, both of which make these species more abundant. However, whether species are perennials or annuals was least important trait determining species abundance. This result may largely attribute to our sampling schemes, and we sampled only once during growth season of most macrophyte species (August to September in 2011). The relative importance of life history traits in determining local species abundance might be increase at larger spatial and temporal scales [30–32].

The relative importance of measured traits (e.g., tuber and leaf nitrogen content, Fig 2e and 2g) in determining species relative abundance varied significantly along water depth gradient, partially supporting our third hypothesis. For these two traits, there were significant shifts in the *λ*-values from positive in shallow water to negative in deep water. This result suggests that macrophyte species with tuber organ and greater leaf nitrogen content would become more abundant in shallow water, but would become less abundant in deep water. The presence of tuber organ may enhance the regeneration of macrophyte species in shallow water, while not necessarily be useful in deep water due to the limitation of low light conditions. Species with high leaf nitrogen content usually show a high photosynthetic rate and fast growth and thus lead to great abundance [33,34]. However, the ecological effects of high leaf nitrogen content on macrophytes may be largely dependent on the water depths or basically light environment in freshwater habitats [11,25,35]. Thus, species possessing favorable traits may become more abundant in benign conditions while less abundant in harsh conditions.

In conclusion, maxent model can be highly robust (99.8%) in predicting the species relative abundance of macrophyte with observed CWM traits as the constraints, while relative low with CWM traits fitted from water depth gradient as the constraints. The measured traits showed notably distinct importance in predicting species abundances, with lowest for perennial growth form and highest for leaf dry mass content. For tuber and leaf nitrogen content, there were significant shifts in the *λ*-values from positive in shallow water to negative in deep water. This result suggests that macrophyte species with tuber organ and greater leaf nitrogen content would become more abundant in shallow water, but would become less abundant in deep water. Our study highlights how functional traits are distributed across gradients provide a robust path towards to predictive community ecology.

## Supporting Information

S1 Table The mean values of 16 traits for the 17 occurred macrophyte species in the studied areas. Leaf [C] indicates leaf carbon content; Leaf [N] indicates leaf nitrogen content; Leaf [C/N] indicates leaf carbon/nitrogen ratio. The types and units of traits were shown in Table 2. The values of three ordinal traits (i. e, floating leaf, perennial growth form and tuber) were 1 (no) and 2 (yes).
(DOCX)
